# [Corrigendum] Long non‑coding RNA HR1 participates in the expression of SREBP‑1c through phosphorylation of the PDK1/AKT/FoxO1 pathway

**DOI:** 10.3892/mmr.2024.13161

**Published:** 2024-01-10

**Authors:** Duan Li, Liwei Guo, Baoguo Deng, Min Li, Tingting Yang, Fan Yang, Zhijun Yang

Mol Med Rep 18: 2850–2856, 2018; DOI: 10.3892/mmr.2018.9278

Subsequently to the publication of the above article, an interested reader drew to the authors’ attention that the data panel for the “Huh7+BSA” experiment shown in Fig. 1D on p. 2852, showing the relative size of lipid droplets as determined in morphological studies using oil red O staining, had also appeared previously in the following article published by the same research group [Li D, Cheng M, Niu Y, Chi X, Liu X, Fan J, Fan H, Chang Y and Yang W: Identification of a novel human long non-coding RNA that regulates hepatic lipid metabolism by inhibiting SREBP-1c. Int J Biol Sci 13: 349–357, 2017]. Upon examining their original data, the authors have realized that this data panel was inadvertently selected incorrectly in Fig. 1, and the revised version of Fig. 1, containing the correct data panel for Fig. 1D, is shown on the next page.

Note that this error did not significantly affect the results or the conclusions reported in this paper. All the authors agree to the publication of this Corrigendum, and are grateful to the Editor of *Molecular Medicine Reports* for allowing them the opportunity to correct this error. Moreover, the authors apologize to the readership for any inconvenience caused.

## Figures and Tables

**Figure 1.**
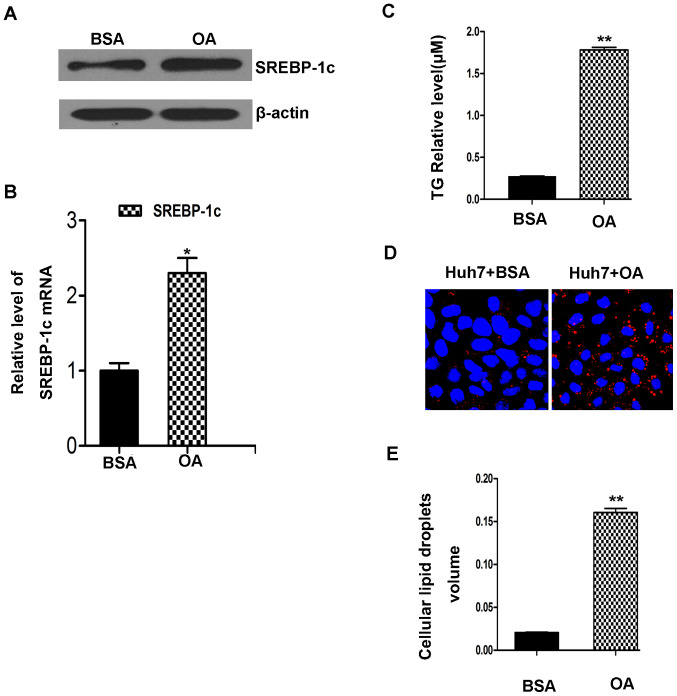
The model of OA induces hepatic cell triglyceride accumulation in Huh7 cells. (A and B) In Huh7 cells, OA treatment for 24 h significantly increased the level of SREBP-1c protein and the expression of SREBP-1c mRNA compared with control cells. (C) OA treatment increased the volume of TG in Huh7 cells. (D) Lipid droplets were significantly increased compared with untreated controls (Scale bar=30 µM). (E) The volume of intracellular lipid droplets was also increased as determined through the quantification column. *P<0.05 and **P<0.01. OA, oleic acid; BSA, bovine serum albumin; SREBP-1c, sterol regulatory element binding protein-1c; TG, triglyceride.

